# *Photobacterium damselae* subspecies *damselae* Pneumonia in Dead, Stranded Bottlenose Dolphin, Eastern Mediterranean Sea

**DOI:** 10.3201/eid2901.221345

**Published:** 2023-01

**Authors:** Danny Morick, Shlomo E. Blum, Nadav Davidovich, Ziv Zemah-Shamir, Eyal Bigal, Peleg Itay, Assaf Rokney, Iris Nasie, Noa Feldman, Marcelo Flecker, Mia Roditi-Elasar, Kobi Aharoni, Yotam Zuriel, Natascha Wosnick, Dan Tchernov, Aviad P. Scheinin

**Affiliations:** University of Haifa, Haifa, Israel (D. Morick, N. Davidovich, Z. Zemah-Shamir, E. Bigal, P. Itay, M. Roditi-Elasar, Y. Zuriel, D. Tchernov, A.P. Scheinin);; Hong Kong Branch of Southern Marine Science and Engineering, Guangzhou, China (D. Morick, D. Tchernov);; Kimron Veterinary Institute, Bet Dagan, Israel (S.E. Blum, M. Flecker);; Israeli Veterinary Services, Bet Dagan (N. Davidovich);; Ministry of Health, Jerusalem, Israel (A. Rokney, I. Nasie, N. Feldman);; Hebrew University of Jerusalem, Rehovot, Israel (K. Aharoni);; Universidade Federal do Paraná, Curitiba, Brazil (N. Wosnick)

**Keywords:** *Photobacterium damselae*, marine mammals, bottlenose dolphin, stranded, *Tursiops truncatus*, pneumonia, *Photobacterium damselae* subspecies *damselae*, bacteria, eastern Mediterranean Sea, zoonoses, Israel

## Abstract

*Photobacterium damselae* subspecies *damselae*, an abundant, generalist marine pathogen, has been reported in various cetaceans worldwide. We report a bottlenose dolphin in the eastern Mediterranean Sea that was found stranded and dead. The dolphin had a severe case of chronic suppurative pneumonia and splenic lymphoid depletion caused by this pathogen.

The common bottlenose dolphin (*Tursiops truncatus*) is perhaps the most common and widespread dolphin species in the Mediterranean Sea (*1*). *Photobacterium damselae* subspecies *damselae* is a pathogen that produces wound infections and hemorrhagic septicemia and high mortality rates and affects various marine animals, such as fish, mollusks, crustaceans, and cetaceans (*2*,*3*). Highly pathogenic *P. damselae* subsp. *damselae* isolates have 2 major virulence factors: the phospholipase D damselysin (Dly) and the pore-forming toxin phobalysin P (initially called HlyA_pl_). Both toxins are encoded by the plasmid pPHDD1 and produce hemolytic and cytolytic activities in a synergistic manner (*4*). We report a bottlenose dolphin in the eastern Mediterranean Sea that was found stranded, dead, and had a severe case of chronic suppurative pneumonia and splenic lymphoid depletion caused by this pathogen.

## The Study

On January 29, 2021, a bottlenose dolphin was found beached nearby Ashdod, Israel. The carcass underwent a postmortem examination based on a widely accepted protocol (*5*) with some modifications because the carcass was also sampled for several anatomic and physiologic studies. Samples of the spleen, liver, lung, kidney and brain were collected for quantitative PCR molecular detection of *Toxoplasma gondii* (*6*) and canine distemper virus (*7*), and for PCR detection of *Brucella* spp. (*8*). Samples of spleen and lung were fixed in 10% buffered formalin for routine histologic evaluation. Samples of lungs and fluid from the thoracic cavity were obtained by using sterile swabs for lung samples and sterile syringes and needles for fluid samples and inoculated onto tryptone soy agar, blood agar (5% sheep blood enriched tryptone soy agar), and MacConkey agar, and incubated for 24–48 h at 37°C. Confirmation of bacteria species was initially performed by using matrix-assisted laser desorption/ionization time-of-flight mass spectrometry according to the manufacturer’s protocol (Autoflex; Bruker, https://www.bruker.com).

**Figure 1:**
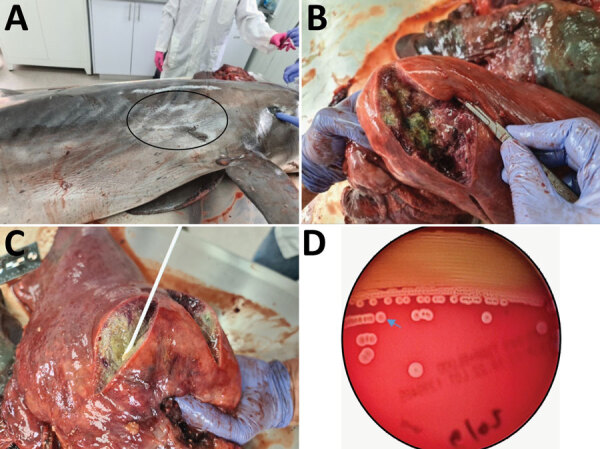
*Photobacterium damselae* subspecies *damselae* pneumonia in a bottlenose dolphin, eastern Mediterranean Sea. Gross pathologic examination of the dolphin (*Tursiops truncatus*) showed a scar (oval) at the right side of the chest (A) that might be a sign for a previous wound that initiated the infection (B, C). Four abscesses, 5–10 cm in diameter, filled with purulent fluid and necrotic debris were observed in the right lung of the animal. Hemolytic phenotype of the *P. damselae* subsp. *damselae* isolate on sheep blood agar (D) indicates the border of the halo of 1 colony (arrow). A weak hemolytic phenotype was observed after culturing isolate on blood agar plates for 24 h.

The dolphin weighed 200 kg, had a length of 263 cm, and was identified as a mature female that had a moderate nutritional status (*9*). At external examination, a deep bruise was observed on the front of the dorsal fin, and an old visible scar was observed on the right side of the chest, which might have been the result of an injury by a foreign body that might have instigated the inflammation within the lung, leading to pneumonia (Figure 1, panel A). No additional external signs of interaction with fishing gear were observed. The carcass was at stage 3 on the decomposition condition code scale (*5*). Internal examination indicated 4 large, firm nodules, 5–10 cm in diameter, replacing the cranial aspect of the right lung lobe. On cut sections, nodules were filled with purulent to caseous, thick, granular, green-tinged exudate surrounded by a dense fibrous capsule (abscess) (Figure 1, panels B, C). No other abnormalities were observed in all other internal organs.

Pure bacterial colonies of spherical or ovoid cocci, 1–2 μm in diameter, consistent with the genus *Photobacterium*, appeared on the blood agar plates at 48-hours postinoculation. Matrix-assisted laser desorption/ionization time-of-flight mass spectrometry confirmed the initial identification of *Photobacterium damselae*. The isolate was resistant to ampicillin and susceptible to gentamicin, sulfamethoxazole/trimethoprim, florfenicol, amikacin, and polymyxin B. The isolate also had intermediate susceptibility to amoxicillin/clavulanic acid; fluoroquinolones; and first-, second-, and third-generation cephalosporins.

The isolate species was also characterized and confirmed by using 16S rRNA gene primers and Sanger sequencing of the 800-nt PCR product. Whole-genome sequencing (WGS) was performed to obtain the allelic multilocus sequence typing (MLST) profile for sequence type determination and to analyze the presence of the 2 *P. damselae* subsp. *damselae* major virulence factor genes (*dly* and *hlyA_pl_*).

We extracted DNA by using the QIAsymphony SP System and the QIAsymphony DNA Mini Kit (QIAGEN, https://www.qiagen.com), according to the manufacturer’s recommendations. We prepared a DNA library by using the Nextera XT Library Preparation Kit (Illumina, https://www.illumina.com), followed by WGS using the Illumina MiSeq and a 250-bp paired-end read length. Reads were assembled by using the BioNumerics 8.0 Platform SPAdes 3.13.1 (Applied Maths, https://www.applied-maths.com).

The assembly was deposited to the pubMLST *P. damselae* database under identification no. 91. We obtained the allelic MLST profile by using the BioNumerics Sequence Extraction Tool (Applied Maths) and according to the *P. damselae* scheme based on 6 housekeeping genes (*glpF*, *gyrB*, *metG*, *pntA*, *pyrC*, and *toxR*) (*10*). This tool was also used for identification of virulence factor gene sequences *dly* (GenBank accession no. 9937366) and *hlyA_pl_* (GenBank accession no. ID 9937197). Hemolysis was tested by culturing the isolate on 5% sheep blood agar (#PD-005; Hylabs Ltd, https://www.hylabs.co.il) for 24 h at 37°C.

Identification of *P. damselae* subsp. *damselae* was supported and confirmed by molecular, phenotypic, and genomic characterization. The 16S rRNA sequence showed a similarity of 99.17% with other *P. damselae* subsp. *damselae* strains in GenBank. When tested for hemolysis, the isolate exhibited a weak hemolytic phenotype, producing narrow halos on sheep blood agar plate (Figure 1, panel D). This phenotype is typical of *P. damselae* subsp. *damselae* lacking the pPHDD1 plasmid and having the chromosomal PhlyC gene (*hlyA_ch_*). WGS of the hemolytic genes *dly* and *hlyA_pl_* yielded only the *hlyA* sequence, which showed 99% identity to the *hlyA_ch_* sequences in GenBank.

The MLST allelic scheme extraction (Table 1) resulted in a new profile that was submitted to the isolate collection of the PubMLST *P. damselae* database as PDIN1, and was assigned a new sequence type (ST), ST63. Within the PubMLST database, most of the *P. damselae* subsp. *damselae* isolates (Table 2) originated from an unusual cetacean mortality event in Italy during 2013 (*11*). Neighbor-joining phylogenetic analysis suggested that the strain from Israel sequenced in this study was not strongly related to any other available ST and showed closest resemblance to isolate ST45 from a bottlenose dolphin from Italy (Appendix Figure).

**Table 1:** Similarity-based gene extraction of genes used in MLST scheme, including the obtained MLST allelic profile for *Photobacterium damselae* subspecies *damselae* pneumonia, in bottlenose dolphin, eastern Mediterranean Sea*

Gene	Identity,† %	Coverage, %	Reference length, bp	No. mismatches	No. open gaps	MLST allelic profiles
*glpF*	99.58	100.00	480	2	0	26
*gyrB*	97.58	100.00	537	13	0	27
*metG*	99.53	100.00	429	2	0	7
*pntA*	97.98	100.00	396	8	0	23
*pyrC*	98.42	100.00	507	8	0	31
*toxR*	93.32	99.74‡	387	23	1	35

*MLST, multilocus sequence typing. †The percentage of identity to allele 1 sequence of each gene (taken from *P. damselae* subsp. *damselae* PubMLST database). ‡The *toxR* alleles length range is 372–390 bp (*10*). The *toxR* allele length of the isolate of this study is 390 bp, and the allele 1 reference length is 387 bp.

**Table 2:** PubMLST database of *Photobacterium* *damselae* subspecies *damselae* isolates from different marine animals, including a bottlenose dolphin in the eastern Mediterranean Sea*

Country	Host	Organ	Year							NA
			2010	2012	2013	2014	2015	2016	2021	
Australia	*Seriola lalandi*	Ot	2	0	0	0	4	2	0	0
Israel	*Tursiops truncatus*	Lu	0	0	0	0	0	0	1	0
Italy	*Caretta caretta*	Icc	0	0	3	0	0	0	0	0
	*Delphinus delphis*	Ot	0	0	1	0	0	0	0	0
	*Physeter macrocephalus*	Br	0	0	0	1	0	0	0	0
		Mf	0	0	0	1	0	0	0	0
		Sp	0	0	0	2	0	0	0	0
		Ut	0	0	0	1	0	0	0	0
	*Stenella coeruleoalba*	Br	0	0	14	3	0	0	0	0
		Icc	0	0	1	0	0	0	0	0
		In	0	0	3	0	0	0	0	0
		Jf	0	0	3	0	0	0	0	0
		Li	0	0	6	0	0	0	0	0
		Ln	0	0	2	0	0	0	0	0
		Lu	0	1	3	0	0	0	0	0
		Ot	0	0	3	0	0	0	0	0
		Sp	0	0	5	0	0	0	0	0
	*Stenella* spp.	Br	0	0	3	0	0	0	0	0
		In	0	0	1	0	0	0	0	0
		Li	0	0	1	0	0	0	0	0
		Ln	0	0	2	1	0	0	0	0
		Lu	0	0	1	1	0	0	0	0
		Sp	0	0	1	0	0	0	0	0
	*Tursiops truncatus*	Br	0	1	3	0	0	0	0	0
		Icc	0	0	1	1	0	0	0	0
		Lu	0	0	1	0	0	0	0	0
		Sp	0	0	1	0	0	0	0	0
		Un	0	0	0	1	0	0	0	0
Japan	*Labracoglossa argentiventris*	Ot	0	0	0	0	0	0	0	1
	*Sardinops melanostictus*	Ot	0	0	0	0	0	0	0	1
United States	*Carcharhinus plumbeus*	Li	0	0	0	0	0	0	0	1
	*Chromis punctipinnis*	Ot	0	0	0	0	0	0	0	1
		Total	2	2	59	12	4	2	1	4

*Database contained 86 isolates as of February 5, 2020 (https://pubmlst.org/organisms/photobacterium-damselae). Br, brain; Icc, intracardiac clot; In, intestine; Jf, joint fluid; Li, liver; Ln, lymph node; Lu, lung; Mf, mesenteric fluid; NA, no year data in PubMLST database; Ot, other; Sp, spleen; Un, unknown; Ut, uterus.

**Figure 2:**
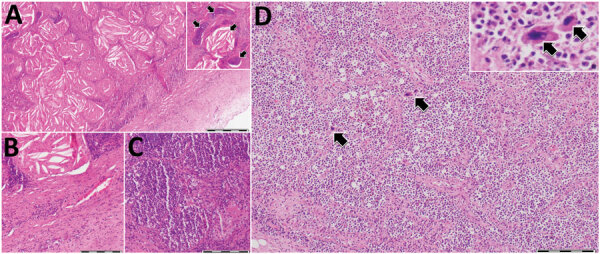
Histologic analysis of lungs and spleen of a bottlenose dolphin (*Tursiops truncatus*) with *Photobacterium damselae* subspecies *damselae* pneumonia, eastern Mediterranean Sea. A) Lung tissue showed a nodular structure covered by fibrous capsule (right bottom of figure panel) composed of numerous cholesterol clefts and areas of reactive fibrosis. Hyaline cartilage was observed, interpreted as bronchi and bronchioles. Inset, higher magnification showing an aggregate of cholesterol clefts and hyaline cartilage (arrow). B) Abundant fibrous lung tissue (lower right half) and cellular infiltrates were also observed. C) Different area of the lung parenchyma characterized by increased cellular infiltration. D) Spleen expressed an apparent contraction of the parenchyma showing diffuse cellularity, with only a few defined lymphoid follicles, as well as megakaryocytes (arrows) indicative of extramedullary hematopoiesis. Inset: higher magnification showing 2 adjacent megakaryocytes (arrows). Hematoxylin and eosin stained. Scale bars indicate 500 μm in panel A and 200 μm in panels B–D.

Results of molecular detection for *T. gondii*, canine distemper virus, and *Brucella* spp. were negative for all tested samples. Examination of lung tissue (Figure 2, panels A–C) showed a nodular structure covered by fibrous tissue composed of extensive cellular infiltration, numerous cholesterol clefts, and areas of reactive fibrosis. A second section of the lung showed extensive tissue lysis and concentric fibrosis of blood vessels. In part of the section, a locally extensive cellular infiltration was observed. An area of necrosis was accompanied by a neutrophilic inflammatory reaction and intralesional bacterial colonies. Two additional tissue sections showed diffuse solid fibrosis, multiple cholesterol clefts, and aggregations of leukocytes. Histopathologic analysis indicated an apparent contraction of the parenchyma with occasional lymphoid follicles and diffuse cellularity within the spleen (Figure 2, panel D), which were suggestive of extramedullary hematopoiesis. Morphologic features of both organs included severe chronic suppurative pneumonia and splenic lymphoid depletion, possibly resulting in extramedullary hematopoiesis in the spleen.

This strain caused severe chronic suppurative pneumonia in the absence of the *dly* gene. This result supports previous indications that this virulence factor is not essential for pathogenesis (*12*).

The antibacterial drug sensitivity test showed susceptibility of the isolate to drugs most frequently used in human and veterinary medicine in this region. Tests results for *T. gondii*, canine distemper virus, and *Brucella* spp. showed negative results, making *P. damselae* subsp. *damselae* the only culturable pathogen identified in the dolphin.

## Conclusions

We report detection of *P. damselae* subsp. *damselae* in a bottlenose dolphin in the Mediterranean Sea. This report adds to the increasing baseline data regarding the health of these marine mammals and provides molecular information for a pathogen capable of infecting a large variety of animals in the marine environment, as well as humans.

Appendix Additional information on *Photobacterium damselae* subsp. *damselae* pneumonia in dead, stranded bottlenose dolphin, eastern Mediterranean Sea.
