# Supplementary material for: Photobacterium damselae subspecies damselae Pneumonia in Dead, Stranded Bottlenose Dolphin, Eastern Mediterranean Sea
Source: Emerg Infect Dis. 2023 Jan;29(1):179–83. doi: 10.3201/eid2901.221345 (PMC9796189; doi:10.3201/eid2901.221345)
Supplement: Appendix — Additional information on Photobacterium damselae subsp. damselae pneumonia in dead, stranded bottlenose dolphin, eastern Mediterranean Sea. [file 22-1345-Techapp-s1.pdf]

Article DOI: https://doi.org/10.3201/eid2901.221345

Photobacterium damselae subspecies *damselae* Pneumonia in Dead, Stranded Bottlenose Dolphin, Eastern Mediterranean Sea

Appendix

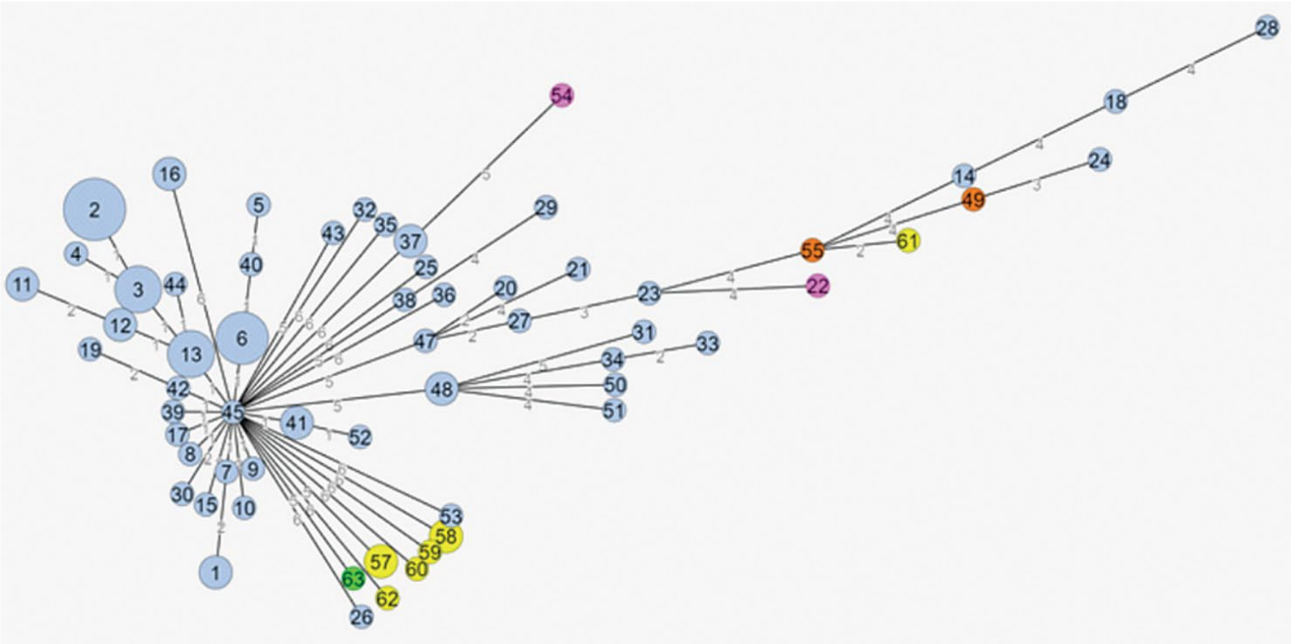

Appendix Figure. Neighbor-joining GrapeTree diagram generated in PubMLST based on the 6-gene multilocus sequence typing (MLST) profiles of all *Photobacterium damselae* subsp. *damselae* strains included in the PubMLST database (https://pubmlst.org/organisms/photobacterium-damselae). Node color indicates the country of origin: Italy (pale blue; 73 strains), Australia (yellow; 8 strains), Japan and United States (orange and pink, respectively; 2 strains each), and Israel (green; 1 strain). Number inside each node indicates sequence type (ST) by the 6-gene MLST scheme of Alba et al. (*1*). Node size is proportional to the number of strains with the same ST. Number within each branch indicates internode allelic distance for ST-63 determined for the *P. damselae* subsp. *damselae* strain characterized in this study in Israel and is shown as a green node.

Reference

1. Alba P, Caprioli A, Cocumelli C, Ianzano A, Donati V, Scholl F, et al. A new multilocus sequence typing scheme and its application for the characterization of *Photobacterium damselae* subsp. *damselae* associated with mortality in cetaceans. Front Microbiol. 2016;7:1656. PubMed https://doi.org/10.3389/fmicb.2016.01656
